# Understanding Different Types of Followers’ Engagement and the Transformation of Millennial Followers into Cosmetic Brand Evangelists

**DOI:** 10.3390/bs13030270

**Published:** 2023-03-19

**Authors:** Warinrampai Rungruangjit, Thitinan Chankoson, Kitti Charoenpornpanichkul

**Affiliations:** 1 Faculty of Business Administration for Society, Srinakharinwirot University, 114 Sukhumvit 23, Bangkok 10110, Thailand; 2 The College of Tourism Hospitality and Sports, Rangsit University, 52/347, Pathumthani 12000, Thailand

**Keywords:** brand community, branded Facebook pages, followers’ engagement, millennial consumers, oppositional brand referral, positive brand referral, purchase intentions

## Abstract

Facebook pages of cosmetic products have substantially grown among millennial consumers. This study aims to explore the motivational factors that affect different types of millennial followers’ engagement, including followers on Facebook pages of cosmetic products, and examine different types of millennial followers’ engagement that influence brand evangelism. A quantitative method involving the technique of partial least square structural equation modeling was applied. An online questionnaire was designed to collect data from millennial followers. The results revealed that informational content stimulates active lurkers and passive participants, while entertaining content positively influences only active participants. Social interaction value is influential to active and passive participants. It was found that identification is the motivation factor that drives both active participants and lurkers. Confidence benefits and special treatment benefits were found to be the motivation factor that stimulates all participants. Social benefits substantially influence active participants and lurkers. Interestingly, the followers’ passive participation has a great influence on brand evangelism. This study opposes the notion that active lurkers and passive participants are less important than active participants and supports the literature by revealing the importance of distinguishing between active participants, active lurkers, and passive participants in causing different impacts on brand evangelism.

## 1. Introduction

Social media has grown tremendously in popularity, with the number of global social media users reaching 4.65 billion in 2022. Social media platforms encourage firms to have direct engagement with consumers and to affect their behaviors regarding products and branded entities [1]. Branded Facebook pages are some of the most popular social media communities, with two billion active monthly users around the world [2]. The number of Facebook users in Thailand is estimated to be more than 50 million, which is equivalent to 78.7% of the total population [3]. With the impressive growth of brand communities in Thailand, various companies are eager to tap into the trend of communicating with customers [4], especially millennials, who have become a key target market for the business sector [5]. The millennial generation is dependent upon social media, particularly for information searches [6] and communicating with their friends and communities [7]. As this generation is quite active in brand communities, they are considered a future consumer market with strong purchasing capacity [8]. Previous studies have indicated that the millennial generation regularly performs purchasing activities with others in virtual communities [9]. They provide and seek comments and credible product information in an enthusiastic way [6]. As a result, both research and industry are increasingly focusing on the effectiveness of digital marketing targeted at this market segment [5].

Interestingly, the cosmetic market is booming and considered one of the most aggressively expanding consumer markets. The generational change, with millennial customers joining the market, is the primary driver of this tremendous growth. At the same time, social media communities, which have a long-term impact on cosmetic product purchasing behavior, also encourage this shift [10]. A study by Cooley and Parks-Yancy [11] found that millennial students used social media communities as information sources for cosmetic products. In Thailand, the expansion of the cosmetic market, internet usage, and online activities has had a great influence on social media communities. In regard to marketing, the cosmetic market has shifted its selling channels because it is more difficult to fulfill customers’ needs and satisfaction using traditional platforms. Currently, Facebook is one of the most effective tools to promote cosmetic items. Branded Facebook pages together with modern shopping behavior are playing more and more important roles in consumers’ daily lives [12].

Branded Facebook pages have altered the role of customers in online communities. Customers have become more alert and engaged both in the purchasing process and other branded activities [13]. It is crucial for businesses to determine how to enhance customer engagement in online communities and define the motivational factors affecting online customer engagement [14]. De Vries and Carlson [15], who conducted their research according to the basic concepts of uses and gratifications theory, specified that brand engagement consists of only three motivating factors (content-oriented, social-relationship-oriented, and self-oriented) without paying attention to relationship benefits. Based on the concept of relational benefits, customers who gain benefits from the relationship are encouraged to build and maintain a long-term relationship with branded entities [16], and customers have to receive benefits from such a relationship so that it will be continued [17]. Long-term customer relationships generate positive outcomes for business entities. Certain outcomes possibly appear in the form of customer engagement [18].

To ensure a long-term relationship, consumers need to perceive the relationship as valuable enough to stay in it. Customers can obviously see some key benefits, such as products, service quality, and prices. Moreover, customers also would like to receive more relationship benefits, including confidence, special treatment, and social benefits [19]. According to past studies, customers’ perceived relationship benefits can increase customer satisfaction and create positive word-of-mouth, and these contribute to attaining the marketing goal of brand communities [20,21]. Nevertheless, most of the current studies have emphasized the relationship benefits with respect to customer–brand relationships in a traditional way, ignoring the prospective relationship benefits gained from customer–other customer relationships on social media platforms [18,22]. However, the study by Andersen [23] focused on the significance of customer-to-customer relationships in online brand communities and suggested that the next study should analyze the potential benefits to be received by members. Many firms are unable to achieve marketing targets through the use of online brand communities, primarily because they do not completely comprehend customer needs for benefits or cannot match customer needs via social media [24]. This study seeks to integrate two primarily literature-based disciplines, uses and gratifications theory and the relational benefits concept, into a consolidated framework in order to close the research gaps.

Previous studies mainly place importance on online followers’ engagement with active participants [25], who are considered better participants than lurkers in online communities [26]. Lurker is normally used to describe someone who observes what is happening but has no participation or remains inactive and is thus related to observation, silence, inactivity, passivity, invisibility, or bystander behavior. Lurkers are described as passive populations who are difficult to reach or get involved in virtual communities [27] and just consume content anonymously [28]. Lurkers are defined as passive participants in this study [29]. In spite of the significance of active user participation in virtual communities, studies show that few people participate in online discussions. According to previous studies, most online community members are lurkers who participate in virtual communities passively [30,31]. Considering the 90-9-1 rule, 90% of online community members simply read posts and do not involve themselves in online discussions; 9% of them edit content and contribute in some way; and only 1% actively participate in online activities and create their own content. Although the exact number differs, it has been concluded that the majority of content in an online community is created by a minority of users [32].

On the contrary, some research studies have shown that passive participants are required as they play an essential role in the community [30]. Even if they simply post occasionally or never, passive participants spend many hours reading information [33]. Alexandrov et al. [34] underlined the significance of passive participants, particularly when the online platform is overloaded with negative data. They indicated that a large number of active participants can be dangerous if the community is dominated by negative remarks since participants’ opinions and purchasing intentions are heavily influenced by what others think. In addition, passive participants with lurking behaviors who only read other people’s comments contribute more to attitudinal and behavioral loyalty to brands than those who share content and provide comments [35]. According to Takahashi et al. [36], a significant number of lurkers utilize and disseminate knowledge and information obtained from online communities in their everyday lives. This type of lurker is known as an active lurker. They came to the conclusion that active lurkers should not be overlooked when assessing the value of online communities at the organizational level, as those lurkers seem to have a powerful and widespread influence in offline communities. Another strategy to add value to online communities is to increase the number of active lurkers [33]. However, most studies on building branding through online communities have paid attention to motivational factors that affect consumers’ active participation in online communities [37] but rarely concentrate on active lurking and passive participation. There is a limited understanding of the nature and interactions of the three characteristics of followers’ engagement on branded Facebook pages. Therefore, the first research question is listed below:

**RQ1: What are the motivational factors that influence different types of millennial followers’ engagement on branded Facebook pages?**

Branded Facebook pages have recently become a crucial element of brand management. However, not every company can build a successful brand page community and maintain good interaction with its customers [38]. This is because companies are likely to place emphasis on the number of comments, likes, and shares, which are considered benchmarks of customer engagement in branded fan pages, without recognizing the actual engagement behavior of their customers [39]. Despite the growing importance of Facebook brand communities, limited research has been conducted to determine how online brand communities contribute to overall brand evangelism, particularly as community members become more involved [40]. Obviously, branded pages are considered a powerful marketing tool that not only affects consumer buying decisions but also brings about brand evangelism and ripple effects [41]. Moreover, social networking fans can efficiently spread positive word of mouth and defend their branded entity [42]. However, many previous research studies have focused on how customer engagement in online communities affects customer satisfaction, commitment, brand trust [43], brand affect, word-of-mouth [44], repurchase intention [45], and brand loyalty [46,47] but overlooked brand evangelism, which is essential in virtual communities because brand evangelism indicates that customers provide full support to the branded entity and build a deep emotional connection with it [48]. Continuing strong relationships through community fan pages is the most effective method of brand marketing management [41]. Firms should create a close connection between the branded entity and consumers and establish customer–brand relationships, which has become more challenging under the current business conditions [49]. It is rather difficult to have an in-depth understanding of brand evangelist behavior, especially on branded Facebook pages. To address this literature gap, the following research question is proposed:

**RQ2: Do the different types of millennial followers’ engagement have an influence on brand evangelist behaviors?**

The following sections provide details about the conceptual background, hypothesis development, and framework of the study. The empirical test of the theoretical framework was conducted with data acquired from an online questionnaire of Thai millennials who have been followers of branded Facebook pages for cosmetics. Each hypothesis in this study was examined by using structural equation modeling (SEM). There is also a discussion on theoretical and managerial implications, as well as limitations and suggestions for future research.

## 2. Theoretical Background and Hypothesis Development

### 2.1. Social Media-Based Brand Community

The concept of a social media-based brand community combines a brand community with social media in a digital setting. According to Schröder and Hölzle [50], the term is defined as a subclass of the overall concept of virtual communities. Brand communities established on a social media platform are referred to as “social media-based brand communities” [51]. Social media is considered an online application platform that enables users to have mutual engagement, cooperate, and share materials with their personal networks. A brand community is described as a specific, geographically boundless community established with a set of structural social relationships between customers and started by enthusiasts [52]. Moreover, a brand community is defined by Stokburger-Sauer [53] as a collection of branded entity lovers who participate in group activities to achieve common goals and express collective ideas and values.

Online brand communities are different from conventional virtual communities because of their standard quality and shared benefits, as well as the compassion, appreciation, and enthusiasm for certain branded entities held by their members [54]. Online brand communities enable their members to directly interact and communicate with one another in order to exchange experiences, feelings, information, and provide one-to-many communication [55] and allow their members to conveniently express personal opinions, which contributes to the establishment of interpersonal relationship networks on the basis of information exchange [56].

Brand communities are part of a social media marketing strategy that provides new ways for businesses and consumers to interact with one another [57]. Companies have perceived the advantages of online brand communities, and these lead to a clearer understanding of consumer experiences, more effective customer communication, and better consumer feedback management [58]. Brand communities do not only provide companies with interactive functions and alternative communication channels but also allow companies to form connections with loyal customers [59], establish long-term bonds with interested members [60], promote brand loyalty, and create good word of mouth [61].

### 2.2. Branded Facebook Pages

Branded Facebook pages are some of the most popular social media-based brand communities. Facebook was launched in 2004 and currently has over two billion active users, enabling users to interact with one another; create their own profiles; invite their family, friends, and coworkers to join their personal networks; send messages; and share content and ideas [62]. Branded Facebook pages allow businesses to connect and communicate with their customers. Businesses should take advantage of branded Facebook pages as a particular marketing tool [63]. Facebook users become fans or followers of a fan page. This preference will appear on their profiles, showing their personal networks that they admire the branded entity of this fan page. Branded Facebook pages are managed by firms, and when a firm builds a fan page, it can post targeted marketing content to build relationships with its target audience [64]. The updated information on this page will also automatically appear on users’ personal news feeds, and they may provide comments, contact the firm, share content on the page, and engage with other followers [63]. Branded Facebook pages enable consumers to interact with the firm in a variety of ways, depending on their public visibility and activeness, which range from somewhat passive and lurking to active and high posting behaviors. Consumers’ publicly accessible interactions may increase the brand’s online visibility [65].

### 2.3. Followers’ Engagement and Participants in Branded Pages

Barger et al. [66] defined online followers’ engagement as posting, sharing, commenting on, and reacting to user-generated material. In addition, Maslowska et al. [67] divided online followers’ engagement behaviors into three categories: observing, participating, and co-creating. The lowest level of followers’ engagement is observing, which is being exposed to brand-related stimuli. Consumers react to stimuli while participating. They are extremely engaged and develop their own content while co-creating [29]. Similarly, Muntinga et al. [68] claimed that there are several levels of online consumer engagement. They developed the consumers’ online brand-related activities (COBRA) model, consisting of three dimensions: consumption, contribution, and the creation of online content. Consumption is the most common sort of activity with the lowest engagement, for example, reading others’ posts without participating. This kind of engagement is called passive participation [69]. Contribution is the medium degree of engagement, covering interactions with brand-related content and communication with others, such as by commenting. Creation is the highest level of engagement, including actively generating firm-related content, using hashtags, and uploading images and video clips [70]. These latter two types are called active participation [68].

The present study classifies branded page participation into three groups: (1) Active participation includes posting messages, sharing posts, advocating, and socializing [71]. Active participants might interact and give feedback to the firm and other followers [2]. (2) Active lurker participation involves silent participants in brand communities, but they share or spread information received from an online community to those outside the community [36]. (3) Passive participation includes reading posts, comments, or reviews and viewing photos without visibly contributing to the online community or outside communities [29]. However, passive participants help keep a balance in virtual communities. In online forums with a large number of participants, while some content creators still need to create content and maintain value, if all participants post or upload content every day, information overload may occur [72]. Passive participants are just as important as posters in online communities that rely on advertising or sponsorship because revenue is based on the number of visitors rather than content posts. Therefore, they should be seen positively and should not be considered insignificant users [27]. If they are misunderstood, it can be harmful to the overall spirit of an online community [33].

### 2.4. Uses and Gratifications Theory

The theory of uses and gratifications (U&G) was introduced by Katz [73] to investigate media effects from the perspective of consumers. It attempts to explain why people use media in different ways [74]. The U&G theory has been used to examine the method and reasons why customers have interactions with media. As the U&G theory posits that consumers are careful and proactive in their media consumption, it is seen as an inventive approach for investigating internet and social media usage because both of them need users’ active engagement [75]. Moreover, the U&G theory is useful in describing users’ incentives and worries about using social media technologies. This theory relies on two assumptions: (1) media consumers are very proactive and make decisions from their past media experiences and (2) media selection and use are purposeful and motivated, with people taking the initiative in choosing and utilizing communication tools to serve their needs in accomplishing personal goals [76]. The most common needs of users can be divided into three categories: (1) a content-oriented area based on media content; (2) a social-relationship-oriented area where users interact with others (such as firms, influencers, and other users); and (3) a self-oriented area based on individual needs [76].

The U&G theory is a popular technique used by academics specializing in technology and media aimed at better understanding people’s goals and motivations in engaging with media [77]. It is also often used to explore the use of new media such as the internet, social networking sites, blogs, and online communities [78]. Many researchers have recently used the U&G theory to analyze consumers’ continuous use of social media, and they have found that gratifications have a substantial impact on users’ continued usage intentions and adoption [79]. For example, Boyd and Ellison [80] studied why individuals use social networking sites. Dholakia et al. [81] investigated why people participate in virtual communities. However, they did not take into account the influence of these gratifications on brand evangelism, limiting the breadth of their findings.

### 2.5. Relationship Benefits

Relationship benefits can be achieved through the relationship marketing strategy. The concept emphasizes that both the consumer and firm need to gain benefits in order to create and sustain the relationship [18]. Perceived relationship benefits initiate a continuous and stable relationship that can help increase consumer satisfaction and generate relationship behavioral outcomes, such as positive oral communication [20], consumer loyalty, increase in revenue, the predictability of sales, cross-sell bundling, increase in customer lifetimes, and customer engagement value [18]. On the contrary, customers need to gain benefits from a long-term relationship in order to ensure a continuous relationship [17]. Customers must see the relationship as valuable enough to stay in it in the long term, and they tend to anticipate additional benefits from these relationships [82]. The term “relationship marketing” was first introduced by Leonard Berry in 1983 [18]. One of the presumptions in marketing is that two parties participating in an exchange should gain benefits from the other side. According to the relational benefits concept, gaining benefits from the relationship enables customers to build and maintain a long-term relationship with firms [16], and customers have to receive benefits from such a relationship so that it will be continued. Long-term customer relationships generate positive outcomes for business entities. Certain outcomes possibly appear in the form of customer engagement [18]. Most of the current studies have emphasized the relationship benefits with respect to customer–brand relationships in a traditional way and ignored the prospective relationship benefits gained from customer–other customer relationships on social media platforms [18,22]. Nevertheless, the study by Andersen [23] focused on the significance of customer-to-customer relationships in online brand communities and suggested that the next study should analyze the potential benefits to be received by members. This study adopted the relational benefits concept through empirical research. Gwinner et al. [17] classified relational benefits into three types: confidence benefits, social benefits, and special treatment benefits.

### 2.6. Brand Evangelist Behaviors

“Evangelist” originates from the Greek term “*Euangelos*”, which means the carrier of good news [83]. The term evangelism is closely linked with Christianity, in which evangelism refers to the proclamation of the Gospel for the purpose of disseminating the teachings of Jesus. The evangelist intends to attract new members to Christianity by preaching in a very passionate manner. As a result, the term “evangelism” is currently used in marketing contexts [84]. The term “brand evangelism” is used to describe a strong consumer–brand relationship involving a great level of word-of-mouth communication [40], which the branded entity adopts to cultivate customers who strongly believe in specific products or services [85]. Although word-of-mouth and brand evangelism share some similarities, brand evangelism is more dominant and extends beyond the sharing of firm-related content [86].

Brand evangelists can act as unofficial brand ambassadors, as they are considered a marketing tool that is more effective than positive word-of-mouth communication [87], and they actively engage with a branded entity [88], have highly emotional relationships, and are truly connected to a firm [89]. Customers who have become brand evangelists are eager to share their passion for their branded entity with other people [86] and actively inform others about their favorable brand experience. They also show strong support for the branded entity by not only sharing good advice but also purchasing products, giving positive feedback, and complimenting the product or branded entity. They strongly desire to endorse the branded entity and persuade others to purchase its products, as well as criticize opposing products and attempt to protect their branded entity [88,90].

This study adopted the behavioral model of Becerra and Badrinarayanan [88], which describes how brand evangelism is characterized by the following three brand-related behaviors: the desire to purchase the branded entity’s products (purchasing intentions), the inclination to praise the branded entity (positive brand referrals), and the proclivity to make negative comments on competing branded entity (oppositional brand referrals).

### 2.7. Research Framework and Formulation of Hypotheses

#### 2.7.1. Relationship between Informative/Entertaining Content and Followers’ Engagement

In technology and media research, the theory of uses and gratifications has been widely used to study consumer motivations and objectives for engagement with media content. According to previous research that used the U&G theory to examine consumers’ engagement in social media and online communities, consumers mainly access online communities to consume informative and entertaining content [77]. Members of social networking sites and branded Facebook pages tend to search for interesting content that is informative and enjoyable [42]. Moreover, entertaining and informative content takes a more significant role than firm-related content in relationships with consumption, creation, and contribution, as it is identified as one of the key motives in online interactions [68].

Cvijikj and Michahelles [91] attempted to explain what motivates individuals to engage with different kinds of content. Previous research has indicated that entertaining and informative content is necessary for followers to engage with social networking sites. Muntinga et al. [68] mentioned that searching for entertaining and informative content promotes online engagement apart from brand-related activities (consumption, creation, and contribution). Tsai and Men [92] described how businesses establish their brand communities on social networks and utilize informative and entertaining content to boost customer–brand relationships and interactions between customers, contributing to improved engagement. Furthermore, according to Kujur and Singh [43], entertaining and informative materials are likely to affect consumer engagement. In particular, entertaining content tends to have the greatest impact on consumer engagement, as it appears to be interesting, attractive, refreshing, and lively. Based on these results, the assumption is that if firms share entertaining content and branded, entity-oriented information on their Facebook brand pages, their needs will be met, contributing to more followers’ engagement. Thus, the following hypotheses were formulated:

**Hypothesis** **1a.** *Informative content has a significant positive impact on followers’ active participation within branded Facebook pages.*

**Hypothesis** **1b.** *Informative content has a significant positive impact on followers’ active lurking participation within branded Facebook pages.*

**Hypothesis** **1c.** *Informative content has a significant positive impact on followers’ passive participation within branded Facebook pages.*

**Hypothesis** **2a.** *Entertaining content has a significant positive impact on followers’ active participation within branded Facebook pages.*

**Hypothesis** **2b.** *Entertaining content has a significant positive impact on followers’ active lurking participation within branded Facebook pages.*

**Hypothesis** **2c.** *Entertaining content has a significant positive impact on followers’ passive participation within branded Facebook pages.*

#### 2.7.2. Relationship between Social Interaction Value and Followers’ Engagement

Socializing with friends and society; requesting assistance and psychological support; and replacing real-life relationships are all examples of social interaction, which is associated with media gratifications [68]. In the realm of social media, a customer must be able to communicate and connect with other customers, feel that they have certain similarities to other customers, and have opportunities to engage with those who are similar to them in order to satisfy the need for social interaction gratification [93]. The research by Daugherty et al. [94] found that social interactions significantly motivate active users to generate content. In addition, Jahn and Kunz [63] discovered an important, positive impact of social interaction value that leads to better engagement on branded Facebook pages. Therefore, this study intends to investigate whether customers with greater perceived social interaction value tend to have better engagement. In order to explore the effect of social interaction value on followers’ engagement, the following hypotheses were formulated:

**Hypothesis** **3a.** *Social interaction value has a significant positive impact on followers’ active participation within branded Facebook pages.*

**Hypothesis** **3b.** *Social interaction value has a significant positive impact on followers’ active lurking participation within branded Facebook pages.*

**Hypothesis** **3c.** *Social interaction value has a significant positive impact on followers’ passive participation within branded Facebook pages.*

#### 2.7.3. Relationship between Identification and Followers’ Engagement

Consumers may want to join a fan page because they want to improve their image or status. By becoming members of a branded page, people set aside values for their own personal identities [63]. According to Algesheimer et al. [95], group engagement is viewed as a demonstration of personal values. Consumers who identify with a branded entity have a sense of belonging, have pride in being a member of the branded entity, are attached to the brand, and share similar passions with other branded page followers. The rise and popularity of social media have provided consumers with new avenues for self-expression, notably through brands [96]. Consumers may join branded Facebook pages to express their personal values and probably use their posts to show their images [63]. When the “like” button is clicked by consumers on a branded Facebook page, it shows up on their branded Facebook pages and adds to their personal profile [29]. When they tag themselves wearing a particular branded entity, their personality and self-expression are reflected through the brand’s image [96]. Moreover, Fernandes and Castro [29] also found that identification positively affects the participation of both active participants and lurkers. Thus, the following hypotheses were formulated to explore the correlation between identification and followers’ engagement:

**Hypothesis** **4a.** *Identification has a significant positive impact on followers’ active participation within branded Facebook pages.*

**Hypothesis** **4b.** *Identification has a significant positive impact on followers’ active lurking participation within branded Facebook pages.*

**Hypothesis** **4c.** *Identification has a significant positive impact on followers’ passive participation within branded Facebook pages.*

#### 2.7.4. Relationship between Confidence Benefits and Followers’ Engagement

Confidence benefits are considered relationship benefits in psychology. These can be perceived by customers when they are creating relationships with a firm. Customers are encouraged to gain more confidence and feel more comfortable when receiving services [17]. Confidence benefits have been studied in the context of brand communities on social media platforms, aiming to explore the effect confidence benefits have on community satisfaction and WOM communication [82].

Confidence benefits represent the confidence and trust customers have in a firm’s generated content on online platforms, causing them to have reduced anxiety when interacting and discussing with others online [18]. Trust is a specific concern in online brand communities [97], and branded entities can achieve an increase in trust as a result of sustainable cooperative relationships in the community [82]. Moreover, trust or confidence benefits potentially affect customer engagement value [18]. Based on the above information, it can be assumed that if a branded entity provides confidence benefits to its branded page followers, their needs will be met, resulting in a higher level of engagement. The following hypotheses were proposed to test this assumption:

**Hypothesis** **5a.** *Confidence benefits have a significant positive impact on followers’ active participation within branded Facebook pages.*

**Hypothesis** **5b.** *Confidence benefits have a significant positive impact on followers’ active lurking participation within branded Facebook pages.*

**Hypothesis** **5c.** *Confidence benefits have a significant positive impact on followers’ passive participation within branded Facebook pages.*

#### 2.7.5. Relationship between Social Benefits and Followers’ Engagement

Social benefits are a type of relationship benefit concept related to a customer being in a friendship with firms or being recognized by them [17]. Social benefits reflect the quality of personal relationships with other members, which include a sense of familiarity and belonging [98]. Social benefits are related to the emotional dimension of relationships, akin to a sense of membership, as all members are supposed to obtain support from the community to which they belong [99]; they are associated with satisfying the social needs of customers and are characterized by friendship and familiarity with other community members [18]. Interacting with a firm on social media fosters a feeling of belonging [100], similar to a sense of membership [101]. The feeling of connectedness and social need fulfillment that online communities supply is considered a crucial benefit [102]. The research by Tourchian et al. [18] found that social benefits can affect customer engagement value. Based on the above information, it can be assumed that if a firm provides social benefits to its brand page followers, their needs will be met, resulting in a higher level of engagement. The following hypotheses were proposed to test this assumption:

**Hypothesis** **6a.** *Social benefits have a significant positive impact on followers’ active participation within branded Facebook pages.*

**Hypothesis** **6b.** *Social benefits have a significant positive impact on followers’ active lurking participation within branded Facebook pages.*

**Hypothesis** **6c.** *Social benefits have a significant positive impact on followers’ passive participation within branded Facebook pages.*

#### 2.7.6. Relationship between Special Treatment Benefits and Followers’ Engagement

Special treatment benefits involve activities that make a customer feel more valuable than other customers. Examples of special treatment benefits include better discounts, faster service, reduced waiting time, and individualized additional services [82]. Online platforms provide marketing managers with the opportunity to deliver customized services to their customers [97], specifically corresponding to customers’ special treatment benefits. Differing from the conventional point-to-point communication scheme, social media confines geographical and material differences and heightens the frequency of social associations [95].

The previous research reported motive for engagement is special treatment benefits or remuneration through sweepstakes [68]. Some studies on social media motivations have suggested that special treatment benefits are a driver of online communities, particularly contributing to them. Remuneration is considered a motivation for follower engagement because a person may use social media in the hopes of receiving a future reward, probably in the form of financial incentives such as money or prizes [103]. Moreover, Cvijikj and Michahelles [91] found that content with engaging activities and competitive rewards leads to higher customer engagement, especially in terms of comments. Based on the above information, the following hypotheses were proposed to test this assumption:

**Hypothesis** **7a.** *Special treatment benefits have a significant positive impact on followers’ active participation within branded Facebook pages.*

**Hypothesis** **7b.** *Special treatment benefits have a significant positive impact on followers’ active lurking participation within branded Facebook pages.*

**Hypothesis** **7c.** *Special treatment benefits have a significant positive impact on followers’ passive participation within branded Facebook pages.*

#### 2.7.7. Relationship between Followers’ Engagement and Brand Evangelist Behaviors

Past studies have shown that consumer engagement in virtual communities deepens relationships and increases loyalty [57]. Customers that participate in the brand community gain a deeper understanding of the firm and form a close bond with it, which might affect their brand loyalty [104]. Consumers’ future behavioral intentions to participate in and recommend the community to others are positively influenced by brand community engagement [95]. Consumers who actively participate in a social networking community are more likely to stay in the community and recommend it to others [105]. Several studies suggest that consumer–brand engagement is associated with positive word of mouth [106] and resistance to negative comments [107]. Similarly, the study by Pornsrimate and Khamwon [5] revealed that consumers who are actively involved in discussions with social media micro-influencers about the firm or branded entity potentially create brand evangelism, including positive brand referrals, purchase intent, and oppositional brand referrals. Likewise, the research by Pongpaew [108] found that active customer engagement from Gen X and millennial individuals on social media platforms has a great influence on purchase intent.

Interestingly, a past study that was conducted on students in a freshman dormitory who utilized and interpreted an interactive email list to facilitate their daily lives showed that lurkers are active readers who discuss mailing list issues in offline modes from time to time. In conclusion, lurkers play a role in creating online communities [36]. According to the study by Fernandes and Castro [29], lurking engagement behavior in virtual brand communities affects brand loyalty more strongly compared with active engagement. Similarly, Shang et al. [35] discovered that passive behavior in virtual communities can help explain brand loyalty, while a consumer’s participation in virtual communities cannot increase brand loyalty. Although posting requires more effort than passive, it is not always associated with a more favorable attitude toward the product or branded entity. This finding implies that community members can distinguish between loyalty to the community and loyalty to the brand and that active involvement is most likely to be related to community loyalty. In addition, Takahashi et al. [36] found that a significant number of lurkers use or propagate knowledge obtained from online communities in their everyday lives. They defined this type of lurker as an active lurker and also concluded that active lurkers should not be overlooked when assessing the value of online communities in an organization since they may have a powerful and extensive influence offline.

Moreover, the study by Rungruangjit and Charoenpornpanichkul [48] found that micro-influencer-generated content on Instagram can affect consumer–influencer engagements consisting of three dimensions: consuming (passive participation), contributing, and creating (active participation). This leads to sustainable consumer–brand relationships, including positive brand referral, purchase intention, and oppositional brand referral. Thus, it is expected that followers’ engagement also evokes brand evangelist behaviors. Thus, the following hypotheses were formulated to explore the correlation between followers’ engagement and brand evangelist behaviors:

**Hypothesis** **8a.** *Followers’ active participation has a significant positive impact on brand evangelism.*

**Hypothesis** **8b.** *Followers’ active lurking participation has a significant positive impact on brand evangelism.*

**Hypothesis** **8c.** *Followers’ passive participation has a significant positive impact on brand evangelism.*

**Figure 1:**
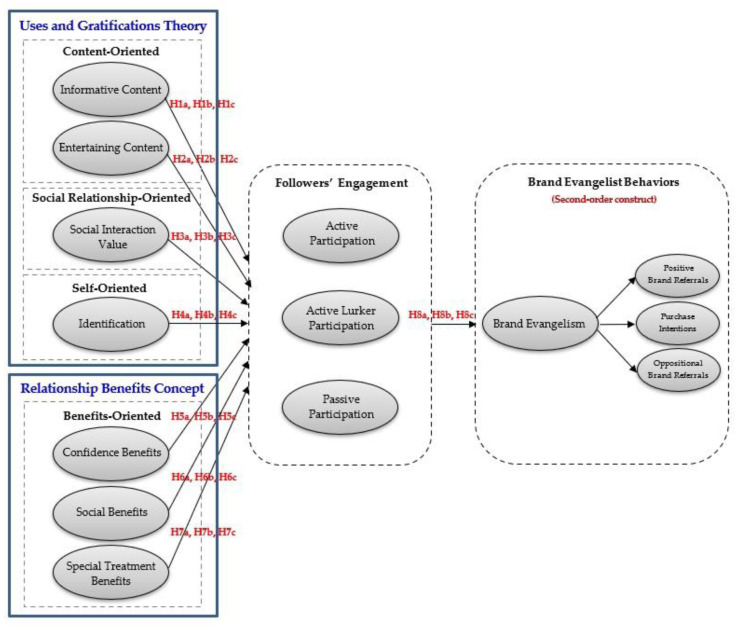
Conceptual framework.

The conceptual framework of this study was established according to the literature review and theoretical framework, as shown in Figure 1.

## 3. Method

### 3.1. Sample Characteristics

The target group included Thai millennials from the ages of 28 to 42 years old (millennial consumers born between 1980 and 1994) [109] who have followed at least one of the top ten most famous Thai cosmetic brands on Facebook (Cute Press, Beauty Cottage, 4U2, Srichand, or Mistine), as rated by Top Best Brand [110]. Millennials are very active on social media and are recognized as a prospective consumer market with strong purchasing power [8]. Facebook was chosen as the social media platform for this research because it is the most popular social media platform, with the maximum number of users in Thailand estimated to be more than 50 million, or 78.70% of the total population [3]. This study focuses on the cosmetics sector since it is prospering and one of the fastest-growing consumer businesses, particularly among millennials [10].

### 3.2. Sample Size

The sample size for PLS-SEM should be calculated by the inverse square root method, as proposed by Kock and Hadaya [111]. As for probability, this method considers that the ratio of a path coefficient and its standard error will be higher than the critical value of a test statistic at a specific significance level. Therefore, the results of the technically required minimum sample size depend only upon one path coefficient, regardless of the size of the most complex regression in the model. The formula for calculating the minimum sample size at a 5% significance level is shown below:

$${\left(\frac{2.486}{\left|Pmin\right|}\right)}^{2}$$

Significance level = 5% : *n*_min_ >

Nevertheless, as researchers have limited data on the expected effect sizes, it is rational to focus more on ranges of effect sizes rather than specific values to finalize the sample size required for a specific study. Therefore, in order to obtain the minimum sample size, the upper boundary of the effect range should be considered a reference because the inverse square root method is quite conservative. In addition, based on a prospective approach, researchers should attempt to determine the minimum expected effect size before conducting data analysis and draw on past studies with concepts or models with comparable complexity [112]. According to a literature review of studies with similar complexity models and contexts, there were six researchers, including De Vries and Carlson [15], Jahn and Kunz [63], Fernandes and Castro [29], Kujur and Singh [76], Pongpaew [108], and Kefi and Maar [113], with a minimum path coefficient ranging between 0.11 and 0.19 with a significance level of 5%. Based on this information, considering the equations, the minimum sample size must not be less than 170, so the appropriate sample size for this study should be between 170 and 510 participants. Thus, a sample size of 510 was chosen.

### 3.3. Data Collection Procedure

In this study, the data were collected by using an online survey. The developed questionnaires were sent to Facebook’s top three online beauty communities in Thailand, namely, Wongnai Beauty, Sistacafe, and Jeban.com [114]. These communities are open online spaces where Thai users come to discuss and share opinions about branded cosmetic entities and products. The following three screening questions were asked to assure that each respondent met the inclusion criteria: (1) Are you between the age of 28 and 42 years old? (2) Are you a follower of any of the following Thai cosmetic brands on Facebook pages: Mistine, Cute Press, Beauty Cottage, Supermom, 4U2, KMA, XOXO, Passion Ville, Cosluxe, or Srichand? (3) Are you a follower of any of the following beauty community Facebook pages: Wongnai Beauty, Sistacafe, or Jeban.com?

The respondents were selected using a probability sampling approach. The stratified sampling method was applied since the population was of a known size. The number of users who have followed the top ten famous Thai cosmetic brands on Facebook was 6,242,683 (data presented as of 15 April 2022). As per the data presented as of 30 June 2022, the three online beauty communities were selected to recruit respondents in this study: (1) Wongnai Beauty, (2) Sistacafe, and (3) Jeban.com. Then, the link to the online questionnaire was publicized on the beauty community’s Facebook pages from May to June 2022. The same Internet Protocol address was allowed to submit data a single time in order to prevent repeated responses. There were 696 returned questionnaires after the end of the data collection. A total of 42 returned questionnaires were removed because of failures to meet the inclusion criteria of the screening questions. In summary, the data returned included 320 responses from the users of Wongnai Beauty, 189 from Sistacafe, and 145 from Jeban.com. In the next step, as per these results, we used a simple random sampling method based on the total number of responses returned in each community to ensure suitable data distribution. Finally, through the analysis of 510 data, we randomized 250 respondents from Wongnai Beauty, 146 respondents from Sistacafe, and 114 respondents from Jeban.com.

### 3.4. Instruments

This research utilized the quantitative method using data acquired from a closed-ended questionnaire to assess the proposed framework. The first section of the questionnaire contained questions for the screening sample. The second section collected personal information. The final section included measurement scales created from previous related studies. Similar to several past studies, this study used a 5-point Likert scale of agreement, ranging from strongly disagree (1) to strongly agree (5) to measure 38 items. The adaptation of the informative content, entertainment content, and social interaction value measurements was performed based on De Vries and Carlson [15] and Gogan et al. [79]. The scale to measure identification was adapted from Vale and Fernandes [115] and Fernandes and Castro [29]. The scale to measure confidence, social, and special treatment benefits was adapted from Zhang and Luo [82] and Tourchian et al. [18]. The scale to measure active and passive participation was adapted from Kefi and Maar [113] and Fernandes and Castro [29]. The scale to measure active lurking participation was modified from that of Takahashi et al. [36]. The scale to measure positive brand referrals, oppositional brand referrals, and purchasing intentions was developed from the studies by Becerra and Badrinarayanan [88], Riorini and Widayati [85], Swimberghe et al. [116], Munasinghe and Dissanayake [117], and Pornsrimate and Khamwon [5].

## 4. Data Analysis and Results

### 4.1. Descriptive Analysis

In this study, the data were acquired from 510 participants. Most of the respondents were female, 355 in total (65.69%), followed by 92 persons of other genders (18.04%) and 83 males (16.27%). Of all the participants, 186 were aged between 28 and 31 years old (36.47%). Regarding academic background, 324 participants (63.52%) graduated with a bachelor’s degree. A total of 330 participants were private company employees (64.71%), and 278 participants earned THB 10,000–30,000 (USD 286.86–860.59) per month (54.51%). An overview of the demographic data of the participants is shown in Table 1.

### 4.2. Data Analysis

Partial least squares structural equation modeling (PLS-SEM) version 3.3.9 was applied to analyze the research model’s relationships, as it has been increasingly emphasized in research over the past period. According to Hair et al. [118], the reason for using PLS-SEM is that it was developed to estimate causal–predictive relationships [119]. PLS-SEM provides explanations and predictions so causal explanations’ practical relevance can be ensured [120]. Compared with covariance-based SEM models, the PLS approach provides advantages in analyzing factorial data as follows: first, PLS imposes less strict assumptions regarding the distributional characteristics of the data; second, it can simply incorporate and model both reflective and formative indicators; third, PLS can best fit with small- and medium-sized samples and can achieve high levels of statistical power with small sample sizes; fourth, it was used for predictive purposes; and fifth, PLS can handle more complicated models that have several structural model relationships [112,121].

### 4.3. Common Method Variance and Nonresponse Bias

Harman’s single-factor test [122] was applied in this study to examine common method variance. To carry out the test, principal component analysis (PCA) was conducted as proposed by Tehseen et al. [123]. According to the unrotated principal axis factoring analysis, a single factor results in 47.127% variance, which is below 50%. The results revealed that every indicator passed the test according to Kock [124]. Therefore, this study has no evidence of common method bias. In other words, it is unlikely to have any major concern that may affect relationships between variables. The common method bias test is shown in Table 2.

To identify the feasible problems of nonresponse bias according to Armstrong and Overton [125], the assessment was made using an extrapolation test to compare means and variance for all constructs in order to examine the difference between the early and late informants, as shown in Table 3. Moreover, to ensure that this research has no problem with nonresponse bias, we conducted an ANOVA test on a set of randomly selected measurement items responded to by both groups of informants. The results reveal that the early and late informants have no significant difference at *p* > 0.05, showing that there is no issue of response bias for the acquired data.

### 4.4. Multicollinearity Test

Multicollinearity exists when two or more correlated predictors in a model show redundant response data. This research tested the multicollinearity between the antecedents of endogenous constructs [126] and demonstrated that the inner VIF is below five, as suggested by Fernández-Portillo et al. [127]. Therefore, there is no multicollinearity in this research. The results are shown in detail in Table 4.

### 4.5. Measurement Model Evaluation

A pre-test was conducted to examine the content validity and reliability of the instruments. To ensure content validity, the assessment of the IOC (index of item–objective congruence) was made by three experts. Feedback on the questionnaire’s format and the vagueness of the questions was provided. The questionnaires were adjusted as deemed necessary. The IOC value must range between 0.50 and 1.00. If the IOC value is below 0.50, a revision will be made as suggested by Rovinelli and Hambleton [128]. Next, 30 sets of the revised questionnaires were tried out to test their reliability. The results reveal that Cronbach’s alpha coefficient ranges from 0.947 to 0.957, meeting the criterion of at least 0.7 [129], and the corrected item–total correlation value ranges from 0.578 to 0.855, meeting the threshold value of at least 0.3 [130].

After the questionnaire passed the pre-test, the next process was to examine the construct reliability. (1) The constructs’ Cronbach alpha (CA) was used to measure the reliability. Since all values are above 0.7, every item exceeded the recommended threshold of 0.7 according to Spira et al. [129]. The results reveal that the variable measurement is reliable and it is not necessary to exclude any item. (2) The composite reliability (CR) of each construct was over 0.8, which was in compliance with the threshold criterion of 0.8 according to Nunnally [131]. In conclusion, the measurements were reliable and accurate. The results are shown in Table 5.

**Figure 2:**
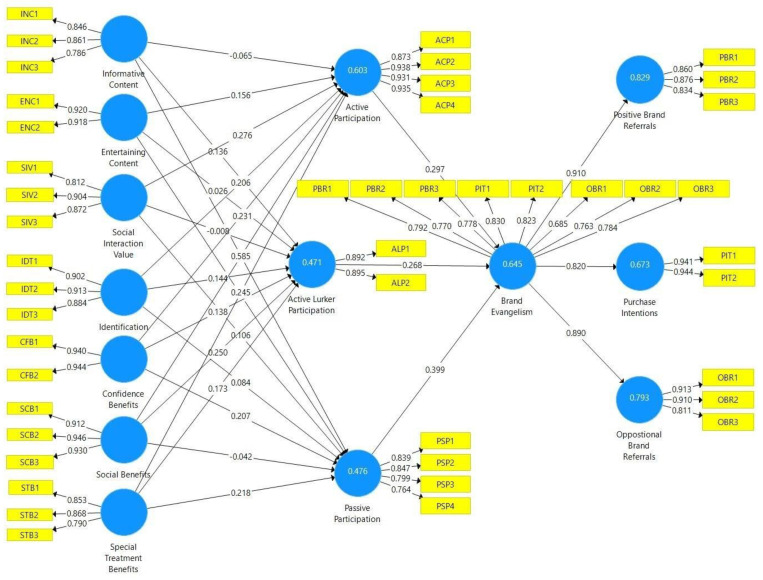
Results of the structural model.

With regard to the convergent validity, (1) the constructs’ items have outer loadings over 0.7, as suggested by Hair et al. [132], and all the constructs’ average variance extracted (AVE) values are over 0.6. All are higher than the suggested threshold of 0.5 according to Fornell and Larcker [133]. Thus, convergent validity is established. The results are shown in Table 5 and Figure 2.

Regarding the discriminant validity, the Heterotrait–Monotrait (HTMT) ratio of correlations technique was applied to assess the discriminant validity. As demonstrated in Table 6, the HTMT correlation ratio of each construct is less than 0.9, which met the threshold value of HTMT [134]. Thus, the discriminant validity of the measurement model was determined.

With regard to the endogeneity issues, a straightforward way of handling, or at least reducing, endogeneity is to specify a set of control variables accounting for a part of the dependent variable’s variance. The Gaussian copula method is particularly popular. The Gaussian copula approach allows researchers and practitioners to detect and correct endogeneity in PLS-SEM [135,136]. This research uses the PLS-SEM algorithm to estimate the model, including the Gaussian copula terms, and determine their significance using bootstrapping. The results are used to assess if critical endogeneity problems exist in the model, and the problems are corrected by the Gaussian copula terms. The results reveal that none of the Gaussian copula values for each construct are significant (*p* > 0.05), suggesting that endogeneity is absent in this study, which meets the threshold requirement [135,137].

### 4.6. Structural Model Analysis

For the predictive model assessment in the PLS-SEM, the researchers conducted a PLSpredict procedure to explore whether the model has a good predictive quality and how much quality it has. A Q^2^ Predict value of zero or below indicates that the predictive power of the PLS-SEM analysis for each indicator does not even surpass the simplest benchmark. For those indicators with Q^2^ Predict values of over zero, the researcher should further compare the RMSE values with the simple LM benchmark [138]. The LM benchmarks are acquired by conducting linear regressions of each of the dependent construct’s indicators on the indicators of the exogenous constructs in the partial least squares path modeling [139]. Based on the test results in Table 7, every indicator has a Q^2^ Predict > 0. When comparing the RMSE values with the LM values, most of the indicators in the PLS-SEM analysis cause smaller prediction errors than the LM. The results reveal a moderate predictive power, based on the guideline proposed by [138].

The results of the structural model are shown in Figure 2. The coefficient of determination, denoted R^2^, is a statistical parameter in a model that specifies the effectiveness of the independent variables in expressing variance in the dependent variable. The R^2^ value for brand evangelism is 0.645, the R^2^ value for active participation is 0.603, active lurking is 0.471, and passive participation is 0.476. Every R^2^ value was in compliance with the threshold criterion of 0.20 according to Cohen [140]. The R^2^ values are shown in Figure 2. The R^2^ value results are the numbers in the circles representing latent variables. Moreover, the correlation between the independent variables and the effect on the dependent variables are expressed by the path coefficient (***β***). The effect sizes of the path coefficient values of this model are shown in detail in Figure 2 Table 8.

### 4.7. Hypothesis Testing

Each path coefficient and hypothesis is shown in Table 8. The results revealed that informative content is significantly influential to both active lurking and passive participation (*β* = 0.136, *t* = 3.134; *β* = 0.261, *t* = 5.950); hypotheses H1b and H1c were accepted. For H1a, the result has no significant influence on active participation (*β* = −0.065, *t* = 1.715). For H2a, entertaining content was found to positively influence active participation (*β* = 0.156, *t* = 4.480); this hypothesis was accepted. In terms of H2b and H2c, the results demonstrated that entertaining content has no significant influence on active lurking (*β* = 0.026, *t* = 0.628) or passive participation (*β* = 0.021, *t* = 0.491). Hypotheses H3a and H3c were supported; the result found that the social interaction value positively affects active participation (*β* = 0.276, *t* = 5.989) and passive participation (*β* = 0.106, *t* = 2.130), while H3b was not accepted, indicating that the social interaction value is not related to active lurking (*β* = −0.008, *t* = 0.179). Hypotheses H4a and H4b were supported; identification is positively related to active and active lurking participation (*β* = 0.206, *t* = 4.698), (*β* = 0.144, *t* = 2.700). On the other hand, H4c was not accepted; identification has no influence on passive participation (*β* = 0.084, *t* = 1.597). Furthermore, confidence benefits significantly influence active participation, active lurking, and passive participation (*β* = 0.231, *t* = 4.422; *β* = 0.138, *t* = 3.239; *β* = 0.207, *t* = 4.331); hypotheses H5a, H5b, and H5c were supported. Social benefits are significantly influential to both active and active lurking participation (*β* = 0.585, *t* = 15.127; *β* = 0.250, *t* = 4.352); hypotheses H6a and H6b were accepted, while for H6c, the result had no significant influence on passive participation (*β* = −0.042, *t* = 0.839). Furthermore, hypotheses H7a, H7b, and H7c were accepted, showing that special treatment benefits affect active participation, active lurking, and passive participation (*β* = 0.245, *t* = 4.455; *β* = 0.173, *t* = 3.335; *β* = 0.218, *t* = 4.584). Finally, active participation, active lurking, and passive participation were found to have positive and significant impacts on brand evangelism (*β* = 0.297, *t* = 9.163; *β* = 0.268, *t* = 6.198; and *β* = 0.399, *t* = 10.500), as hypotheses H8a, H8b, and H8c were supported. 

## 5. Discussion and Implications

### 5.1. Discussions

This research examines the role of motivational factors that influence different types of millennial followers’ engagement, including active participation, active lurking, and passive participation, on branded cosmetics Facebook pages and investigates the effect of different levels of millennial followers’ engagement on brand evangelist behaviors, reflecting a long-term follower–brand relationship. A branded fan page can help members form and maintain long-term relationships with a branded entity [60]. Thus, this is a challenging topic for marketers who have increasingly focused on how to build and nurture successful brand communities.

This study provides evidence that informative content does not influence followers’ active participation on branded pages, which seems to be inconsistent with hypothesis H1a. This finding differs from past research by Kefi and Maar [113], but it reveals an interesting new aspect of millennial consumers’ active behavior. In the case of new products, informative content is important because it provides consumers who are active users with new information about the branded entity. However, once the branded entity becomes well known, active users are likely to lose interest in it [40]. Informative content is not as effective as interactive posts in promoting active engagement on branded pages since active users’ interactions with posts help produce experiential value. Meanwhile, informative content positively influences followers’ active lurking participation (H1b); this finding extends previous research by Kefi and Maar [113] and Fernandes and Castro [29]. Active lurkers use active participants’ ideas and opinions about cosmetics as a source of inspiration and propagate the obtained information to their friends or use that information in their daily routines. Furthermore, the finding revealing that informative content was a powerful motivating factor in followers’ passive participation (H1c) supports the previous research by Kefi and Maar [113] and Fernandes and Castro [29]. Passive users browse online to look up product/branded technical specs and learn about what is new and trendy. Knowledge about the product straightforwardly indicates that passive users consume branded-entity information to better understand the product. Passive users prefer informative content because they want to learn everything about the product, and this satisfies their desire to keep up with current activities about new and existing products. Passive users realize the pros and cons of a product and gain access to inaccessible data because of salesmen’s bias [68].

The second finding revealing that entertaining content has a positive influence only on followers’ active participation (H2a) supports the previous research by Kefi and Maar [113]. Millennial consumers use branded community pages for leisure and entertainment purposes. Entertaining content appears to have an influence on consumer engagement since it is funny, delightful, exciting, and lively. Active users emphasize that pleasing and amusing content arouses their attention, while entertaining content does not significantly affect followers’ active lurking and passive participation (H2b, H2c), which is consistent with the study by Vale and Fernandes [115]. This might be because page-users primarily pay attention to the community to which they belong, and entertaining content is irrelevant. This finding may imply that active lurkers and passive users are more prone to searching for information with utilitarian rather than hedonic values.

The third finding indicating that the social interaction value is the motivational factor driving active participation (H3a) supports previous research by Jahn and Kunz [63] and De Vries and Carlson [15]. Active users would like to meet likeminded users, interact and talk with these users about specific products, and share certain interests. Active users in online brand communities who have built long-term relationships, with the branded entity known as the common denominator, start to have technique-based conversations and become a big circle of friends, providing mutual assistance to each other even though most of these users have not met face-to-face with one another [68]. In the meantime, the social interaction value is also significantly influential to passive participants (H3c), but the results are inconsistent with Jahn and Kunz [63] and De Vries and Carlson [15]. Although these people do not interact with other members, they make friends with those who share the same interests. They would like to meet similar people on branded cosmetics Facebook pages in order to obtain information from people with similar preferences to them. For active lurkers, the finding revealing that the social interaction value insignificantly influences followers’ active lurker participation (H3b) contributes to a new body of knowledge. Social interaction value is difficult to obtain through active lurking participation, which might help explain this finding. According to Shao [141], when users are motivated by self-expression or social reasons, they will focus less on informative content and will only read posts or product reviews for self-related purposes.

The fourth finding suggesting that identification is the motivational factor of followers’ active participation (H4a) supports the research results of Pagani et al. [142], as well as Fernandes and Castro [29]. Active users may join a branded fan page because they want to use their posts to depict their own images, and they upload pictures or create content because they are very curious about other members’ reactions. They share information to familiarize other members with a product. Active users can confirm that they are part of a specific community of brand lovers by posting the branded entity’s content [68]. If active users tag a branded page in their posts, the brand’s image may reflect their personality and allow them to express themselves [93]. Identification also influences followers’ active lurking participation (H4b). This finding extends the current body of knowledge on the behavior of active lurkers. Active lurkers may join a branded fan page to express their personal values. They want to impress their friends with what they know about the cosmetics product. However, the self-expression of this group is different from that of active users. They tend to propagate information or content received from cosmetics’ branded pages to those outside their virtual communities. The identification factor does not impact passive participation (H4c), and this finding supports the previous research by Pagani et al. [142], as well as Vale and Fernandes [115]. According to Nonnecke et al. [143], passive users neither want to impress other members nor participate in group activities because they have more to learn about the group, are shy about posting, or still have nothing to offer. As a result, marketers may not know how to motivate or incentivize them. Moreover, marketers may overlook this segment and focus more on active users, leaving them feeling disconnected from the branded pages.

The fifth finding indicates that confidence benefits contribute to driving active participation, active lurking, and passive participation (H5a, H5b, H5c). This finding extends the current body of knowledge concerning the relationship benefits concept in the context of branded community pages. In terms of confidence benefits, open discussions and interactions on a branded community page encourage active users and active lurkers to have confidence in the credibility of the branded entity and the accuracy of its content, which finally motivates them to engage with brand-related activities and share information or content with their friends in real life. For passive users, if they feel confident in the correctness of a firm’s generated content on online platforms, they will have reduced anxiety when interacting and discussing with other online members and continue consuming the content in the long run.

The sixth finding established the positive effect of social benefits on active participation and active lurker participation (H6a, H6b). This finding extends the current knowledge of relationship benefits for branded Facebook pages. Social benefits specify the strength of personal ties with other members and brands, and this is associated with a sense of belonging and familiarity. The emotional aspect of relationships is reflected in social benefits. According to Teng [99], social benefits are equivalent to a sense of membership, as all members can obtain assistance from their communities. On the contrary, this research hypothesized a positive impact between social benefits and passive participants (H6c), but it was an unsupported hypothesis. With regard to passive users, they silently consume content on branded pages without familiarity with other members. Moreover, they do not want to show their identity and gain recognition from some members on the branded pages, so they are clearly different from the active users.

Furthermore, H7a, H7b, and H7c, which proposed the positive influence of special treatment benefits on active participants, active lurkers, and passive participants, were supported. This finding extends the current body of knowledge concerning the relationship benefits concept in the context of branded Facebook pages. Special treatment benefits also play an important role in encouraging followers to engage with branded Facebook pages. Passive users who are provided with special treatment benefits can even change from consumers to contributors. Remuneration is considered a motivation for passive or consumption users. Even passive users who receive this motivation can change from consuming to contributing users. Remuneration was found to be a motive for passive or consumption users [68].

Finally, followers’ active participation, active lurking participation, and passive participation were found to have a positive impact on brand page evangelism (H8a, H8b, H8c). This finding extends the current body of knowledge about brand evangelism in Facebook communities. Many research studies have suggested that followers’ active engagement in brand communities has an influence on brand loyalty and consumer–brand relationships [57,104]. Interestingly, this study found that followers’ passive participation has the greatest influence on brand evangelism. Passive followers and active lurkers should be regarded as positive participants rather than free riders. They are very active readers, occasionally discuss the branded entity’s content with their real-life friends, and have an intimate relationship with the branded entity. They are willing to endorse the branded entity, persuade others to purchase the brand’s products, defend the branded entity, and make negative comments about other branded entities.

### 5.2. Theoretical Contributions

This study intends to add findings to the existing body of knowledge by exploring the motivational factors that influence followers’ engagement on branded cosmetics Facebook pages and consider whether they lead to brand evangelism behaviors. Academia can benefit from the findings of this research in the following ways. First, prior studies conceptualize that there are two constructs of participation: active and passive [15,29,37,43,63,103,113], but this article expands the body of knowledge by proposing three participation dimensions (active, active lurking, and passive) [36].

This study debunks the notion that active lurking and passive participation are less valuable than active participation. The findings of this research contribute to the literature by showing the importance of distinguishing between active participation, active lurking, and passive participation to determine the differential impact of the three participation dimensions on brand evangelism. While some studies ignore passive participation [15,63]; convey a negative attitude toward passive participants [143]; and overlook active lurkers [29,43,113], the results of this study reveal that active participation, active lurking, and passive participation are all important in fostering brand evangelism. Passive participation in particular was found to be a stronger driver than active participation. Thus, marketers should not disregard passive participants and active lurkers but should pay more attention to them.

Furthermore, the findings of this study contradict delurking strategies [144,145]. Many researchers have tried to minimize the number of lurkers in online communities by employing delurking strategies that encourage lurkers to turn into active posters. Delurking might not always be the most effective way to enhance the value of online communities. An increase in active posters could result in information overload [27]. Attempting to convert lurkers into posters or active users may fail to establish a strong emotional bond with a firm. Taking a role as indirect contributors, lurkers can aid online communities by spreading a firm’s content to other communities and using the obtained information in real-life activities. Thus, converting a lurker to an active user is not necessarily a critical task. An increase in active lurkers can also add value to online communities and lead to brand evangelism.

In addition, this research contributes to the existing body of knowledge by highlighting the importance of the relationship benefits concept in the context of brand communities for the first time. Previous research on brand communities largely relied on the uses and gratification theory [15,29,43], which focuses on the main motivation drivers of consumer engagement (including content-oriented, social-relationship-oriented, and self-oriented engagement) but overlooked relationship benefits factors. Receiving benefits from the relationship causes customers to establish and retain a long-term relationship with firms. Many firms struggle to leverage online brand communities to achieve marketing objectives but lack an understanding of how to effectively satisfy customer benefit needs in online communities. The results of this study demonstrate that relationship benefits have an influence on followers’ engagement. Therefore, it is beneficial to theoretically combine two primarily literature-based disciplines, the theory of uses and gratifications and the concept of relationship benefits, into a unified framework.

Finally, in previous research, they focused on studying followers’ engagement in online communities that influences customer satisfaction, commitment, brand trust, brand affect, word-of-mouth, or brand loyalty. However, this research places an emphasis on brand evangelism behaviors, which are vital in online communities, because brand evangelism reflects the ways consumers strongly embrace a branded entity and develop a profound emotional bond with that branded entity. Brand evangelists can be seen as having an advanced level of positive word-of-mouth because they intend to convince others to buy the branded product, deter others from buying competing branded products, and may even degrade the competitors of their cherished branded entities.

### 5.3. Managerial Implications

This research has important managerial implications concerning social media marketing tactics and will benefit marketers all around the world. The findings of this study will be extremely useful to marketers as they highlight motivational factors that promote followers’ engagement as well as the benefits of brand evangelism on cosmetics’ branded Facebook pages. Marketers’ ability to anticipate and increase followers’ engagement is based on their understanding of such motivational factors. The findings of this study can be utilized to create campaigns that promote various levels of interaction from followers, including active participation, active lurking, and passive participation, in the following ways. First, as informative content has an influence on the active lurking and passive participation of millennial followers, marketers should regularly provide useful information and a variety of content, such as cosmetic ingredients and features, product pros and cons, cosmetic trends, what is new, what is next, upcoming events, and other creative materials, in order to convince active lurkers and passive users that their products are worth purchasing.

Second, upon recognizing that entertaining content has an effect on millennial followers’ active participation, marketers should use a wide range of entertaining content, such as jokes, puzzles, puns, fortunetelling, performances by brand endorsers, and emotionally appealing stories, to advertise their new and existing products or services in order to encourage active users to engage in brand-related activities. Entertaining content will encourage active users to share posts or leave comments, resulting in brand evangelism behaviors.

Third, active participants place more emphasis on the social interaction value than active lurkers and passive participants. Therefore, marketers should provide this group of users with the opportunity to communicate with other members continually. Marketers should encourage active users to upload their generated content. For example, marketers pay only members who create high-quality posts for their ability to generate comments and shares. Such interactivity superseding membership can be implemented in two ways. Members can gain income from every view of their posts and the number of people who share their posts once they are considered high quality. By doing so, members are more likely to receive special treatment from the branded entities.

Fourth, identification is a factor that motivates both active participation and active lurking participation. Marketers should develop challenging activities that drive active users to generate their own content, such as product reviews and recommendations, or challenge active users to produce short video clips that reflect their personalities and self-expression through various campaigns in order to draw more comments and shares on branded pages. In terms of active lurkers, marketers may create activities to encourage active lurkers to buy more products and write an article or blog post to spread content from branded cosmetics entities to their friends, colleagues, and relatives and provide them with special discounts. All related activities must be designed to make users feel proud of the branded entity and feel a sense of belonging when they can impress other members with what they know about the cosmetic product, which contributes to long-term follower–branded entity relationships and reflects brand evangelism behaviors.

Furthermore, active users, active lurkers, and passive users are more concerned about information because they intend to seek maximum details about the product. Thus, marketers should make them feel confident in the accuracy and trustworthiness of the product’s informative content and willing to disseminate the information or content to their friends in real life. In addition, regarding social benefits, marketers should focus on building friendship and familiarity between members of the branded page’s community, especially the active users. On special occasions, marketers may organize fun activities joined by the active users and other members of the branded pages or hold an online event or creative offline event activities on various occasions to help strengthen the friendship between the group members. Lastly, as for special treatment benefits, marketers should find ways to increase special treatment benefits for followers, such as with better or faster services, personalized services, special deals and discounts, monetary rewards, giveaways, or other kinds of prizes when they interact with the branded entity, other members, or brand influencers. Marketers may need to consider the tailored use of special treatment benefits for each segment of the brand community’s members. This is considered an effective way to motivate users to develop strong personal relationships with the branded entity and other members.

Finally, marketers should not only place importance on active users but should also design supporting activities and content to promote the participation of active lurkers and passive users. This is because active lurkers and passive users are vital to online communities and seem to have a certain degree of influence in offline settings. Passive participation contributes more to brand evangelism than active participation. Different types of users exist in online brand communities depending on how close they are to the firm and the other members. Not all consumers interact with firms in the same manner. To build brand evangelism, marketers must first understand the motivational factors that influence distinct followers’ engagement behaviors. Once a customer turns into a brand evangelist, they are eager to act as a brand’s unpaid spokesperson, actively disseminate favorable brand experiences to their friends, urge others to buy the same branded product, and discourage others from buying competing branded products. As a result, it is a difficult task for competitors to attract their attention.

### 5.4. Limitations and Future Research

The limitation of this research relates to the use of a closed questionnaire as opposed to more open methods. There are pragmatic reasons why this might be required; however, it does mean the results may be influenced by self-generated validity [146], where participants create opinions as a result of participating in the study as opposed to study measurement opinions that already existed in the participants. Future research may want to use qualitative techniques to validate the findings and help explain the mechanisms behind the relationships that were found. In addition, the current research solely focused on Thai millennial users. Therefore, the next study should study the behavior of consumers in other generations in different countries. Moreover, this study concentrated on only one famous social networking site: Facebook. Other prominent social networking sites, such as Instagram or Twitter, should be included in future studies because their aims and features are distinct. This would provide a more comprehensive picture of consumer participation and brand evangelism. Finally, this research placed emphasis on firm-generated content, including both informative and entertaining content. Influencer-generated content and consumer-generated content should be explored in further studies.

## Figures and Tables

**Table 1:** Demographic characteristics of the respondents (*n* = 510).

Variable	Category	Frequency	Percentage
Gender	Female	335	65.69
	Male	83	16.27
	Other genders	92	18.04
Age	28–31 years old	186	36.47
	32–35 years old	153	30.00
	36–39 years old	89	17.45
	40–42 years old	82	16.08
Education level	Below bachelor’s degree	34	6.67
	Bachelor’s degree	324	63.52
	Master’s degree	136	26.67
	Doctoral degree	16	3.14
Occupation	Government officers/employees	109	21.37
	Students	46	9.02
	Private company employees	330	64.71
	Business owners	25	4.90
Income	<THB 10,000 (<USD 286.86)	24	4.71
	THB 10,000–30,000 (USD 286.86–860.59)	278	54.51
	THB 30,001–50,000 (USD 860.59–1434.31)	115	22.54
	>THB 50,000 (>USD 1434.31)	93	18.24

Note: 1 USD equals 34.86 THB (the exchange rate as of 2 March 2023).

**Table 2:** Common method variance via the single-factor test.

Factor	Initial Eigenvalues			Extraction Sums of Squared Loading		
	Total	% of Variance	Cumulative %	Total	% of Variance	Cumulative %
1	20.164	47.127	47.127	20.164	47.127	47.127
2	3.828	8.947	56.074			
3	1.949	4.555	60.629			
4	1.363	3.187	63.816			
5	1.192	2.787	66.603			

Extraction method principal axis factoring.

**Table 3:** Nonresponse bias.

	Sample Different Test	
Constructs	Mean	Variance
Active lurker participation (ALP)	0.0774	0.4426
Active participation (ACP)	0.1544	0.4442
Brand Evangelism (BEV)	0.0953	0.6462
Confidence benefits (CFB)	0.2455	0.6556
Entertaining content (ENC)	0.1646	0.5333
Identification (IDT)	0.0744	0.1466
Informative content (INC)	0.0936	0.6242
Passive participation (PSP)	0.3663	0.8559
Social benefits (SCB)	0.1125	0.1854
Social interaction value (SIV)	0.0787	0.4445
Special treatment benefits (STB)	0.0956	0.6342

**Table 4:** Collinearity statistics (inner VIF values).

	ALP	ACP	BEV	CFB	ENC	IDT	INT	OBR	PSP	PBR	PIT	SCB	SIV	STB
ALP			2.246											
ACP			1.648											
BEV								1.000		1.000	1.000			
CFB	1.853	1.853							1.853					
ENC	1.856	1.856							1.856					
IDT	2.022	2.022							2.022					
INT	2.045	2.045							2.045					
OBR														
PSP			1.616											
PBR														
PIT														
SCB	2.487	2.487							2.487					
SIV	1.974	1.974							1.974					
STB	2.062	2.062							2.062					

**Table 5:** Construct reliability and convergent validity.

Construct	Items	Loading	CA	CR	AVE
Informative content (INC)			0.777	0.870	0.691
	INC1: The content on cosmetics’ branded Facebook pages is helpful to me.	0.846			
	INC2: The content on cosmetics’ branded Facebook pages is practical.	0.861			
	INC3: The content on cosmetics’ branded Facebook pages contains a wide range of information.	0.786			
Entertaining content (ENC)			0.816	0.916	0.845
	ENC1: The content of cosmetics’ branded Facebook pages is enjoyable.	0.920			
	ENC2: The content on cosmetics’ branded Facebook pages is pleasant.	0.918			
Social interaction value (SIV)			0.892	0.898	0.745
	SIV1: I can meet people who are similar to me on cosmetics’ branded Facebook pages.	0.812			
	SIV2: I can meet new people who are similar to me on cosmetics’ branded Facebook pages.	0.904			
	SIV3: I can communicate with people who are similar to me on cosmetics’	0.872			
	branded Facebook pages.				
Identification (IDT)			0.882	0.927	0.809
	IDT1: I have a sense of connectedness to cosmetics’	0.902			
	branded Facebook pages.				
	IDT2: I am proud to be a member of cosmetics’ branded Facebook pages.	0.913			
	IDT3: I want to make my friends and others impressed with my	0.884			
	knowledge of cosmetic brands.				
Confidence benefits (CFB)			0.873	0.940	0.887
	CFB1: I feel more confident in the correctness of the content on	0.904			
	cosmetics’ branded Facebook pages.				
	CFB2: I can trust the content of cosmetics’ branded Facebook pages.	0.944			
Social benefits (SCB)			0.921	0.950	0.864
	SCB1: I am recognized by certain members in	0.912			
	cosmetics’ branded Facebook pages.				
	SCB2: I am familiar with other members on	0.946			
	cosmetics’ branded Facebook pages.				
	SCB3: I have formed friendships with certain members on	0.930			
	cosmetics’ branded Facebook pages.				
Special treatment benefits (STB)			0.787	0.876	0.702
	STB1: I occasionally receive discounts or special offers that other	0.853			
	customers do not receive on cosmetics’ branded Facebook pages.				
	STB2: I get better and faster services.	0.868			
	STB3: I get faster answer questions and service.	0.790			
Active participation (ACP)			0.939	0.956	0.846
	ACP1: I often like cosmetic content on branded Facebook pages.	0.873			
	ACP2: I often share posts of cosmetic content on branded Facebook pages.	0.938			
	ACP3: I often comment on cosmetic content on branded Facebook pages.	0.931			
	ACP4: I participate in brand-related discussions on branded Facebook	0.935			
	pages.				
Active lurker participation (ALP)			0.748	0.888	0.799
	ALP1: I share information or content obtained from cosmetics’	0.892			
	branded Facebook pages to those outside it.				
	ALP2: I have used information or content obtained from	0.895			
	cosmetics’ branded Facebook pages for my own activities.				
Passive participation (PSP)			0.829	0.886	0.661
	PSP1: I frequently read posts published on cosmetics’ branded	0.839			
	Facebook pages.				
	PSP2: I frequently watch photos or videos published on	0.847			
	cosmetics’ branded Facebook pages.				
	PSP3: I frequently read the comments of other followers on	0.799			
	cosmetics’ branded Facebook pages.				
	PSP4: I frequently read reviews cosmetics products on	0.764			
	cosmetics’ branded Facebook pages.				
Brand evangelism (BEV)—*Positive brand referral (PBR)*			0.818	0.892	0.734
	PBR1: I use word of mouth to disseminate positive news about the	0.860			
	cosmetic branded entities that I follow.				
	PBR2: I recommend my friends use cosmetic branded entities that I follow.	0.876			
	PBR3: If my friends were seeking cosmetics, I would recommend the	0.834			
	cosmetic branded entities that I follow.				
Brand evangelism (BEV)—*Purchase intentions (PIT)*			0.875	0.941	0.889
	PIT1: I am most likely to buy cosmetic branded entities that I follow.	0.941			
	PIT2: I am willing to buy products from cosmetic branded entities that I follow.	0.944			
Brand evangelism (BEV)*—Oppositional brand referral (OBR)*			0.852	0.911	0.773
	OBR1: If someone tries to say a negative comment about cosmetic branded	0.913			
	entities that I follow, I will correct them right away.				
	OBR2: If someone tries to criticize the cosmetic brands that I follow, I will	0.910			
	directly reprimand them.				
	OBR3: When my friends are shopping for cosmetics, I will advise them to	0.811			
	avoid other brands.				
Brand evangelism (second-order construct)			0.907	0.925	0.607
	Positive brand referral	0.910			
	Purchase intentions	0.820			
	Oppositional brand referral	0.890			

CA = Cronbach’s alpha, AVE = average variance extracted, CR = composite reliability.

**Table 6:** Discriminant validity using Heterotrait–Monotrait (HTMT).

	ALP	ACP	BEV	CFB	ENC	IDT	INC	OBR	PSP	PBR	PIT	SCB	SIV	STB
ALP														
ACP	0.748													
BEV	0.752	0.678												
CFB	0.627	0.393	0.669											
ENC	0.563	0.560	0.618	0.491										
IDT	0.734	0.705	0.788	0.646	0.652									
INC	0.633	0.406	0.711	0.629	0.775	0.671								
OBR	0.746	0.776	0.855	0.545	0.586	0.826	0.610							
PSP	0.774	0.437	0.781	0.657	0.519	0.613	0.704	0.611						
PBR	0.782	0.621	0.838	0.643	0.601	0.724	0.667	0.840	0.783					
PIT	0.674	0.390	0.789	0.648	0.477	0.555	0.669	0.673	0.753	0.776				
SCB	0.691	0.807	0.672	0.471	0.562	0.801	0.468	0.755	0.459	0.616	0.407			
SIV	0.598	0.506	0.635	0.567	0.502	0.735	0.616	0.603	0.585	0.597	0.516	0.690		
STB	0.731	0.564	0.731	0.719	0.530	0.743	0.614	0.669	0.689	0.678	0.639	0.674	0.645	

**Table 7:** Model’s predictive power.

Indicators of	PLS-RMSE	Q^2^_Predict_	LM-RMSE	Diff
ALP1	0.905	0.334	0.908	−0.003
ALP2	0.959	0.390	0.970	−0.011
ACP1	1.002	0.426	1.019	−0.017
ACP2	0.961	0.531	0.963	−0.002
ACP3	0.996	0.506	1.013	−0.017
ACP4	0.945	0.540	0.961	−0.016
OBR1	0.909	0.335	0.912	−0.003
OBR2	1.001	0.424	1.015	−0.014
OBR3	1.037	0.379	1.043	−0.006
PBR1	0.866	0.404	0.852	0.014
PBR2	0.856	0.331	0.862	−0.006
PBR3	0.783	0.298	0.768	0.015
PIT1	0.688	0.358	0.665	0.023
PIT2	0.958	0.399	0.977	−0.019
PSP1	0.763	0.362	0.760	0.002
PSP2	0.827	0.339	0.835	−0.008
PSP3	0.919	0.279	0.934	−0.015
PSP4	0.762	0.226	0.749	0.013
Second-order construct				
OBR1	1.042	0.338	1.070	−0.028
OBR2	0.665	0.304	0.717	−0.052
OBR3	0.720	0.321	0.691	0.029
PBR1	0.853	0.408	0.864	−0.011
PBR2	0.858	0.326	0.859	−0.001
PBR3	0.790	0.286	0.767	0.023
PIT1	0.804	0.429	0.820	−0.016
PIT2	0.794	0.437	0.814	−0.020

**Table 8:** Path coefficients and hypothesis testing.

Hypotheses	Path	Path Coefficients (*β*)	*t* Statistic	*p*-Value	Results
H1a	INC → ACP	−0.065 ^ns.^	1.715	0.087	Not Supported
H1b	INC → ALP	0.136 **	3.134	0.002	Supported
H1c	INC → PSP	0.261 ***	5.950	0.000	Supported
H2a	ENC → ACP	0.156 ***	4.480	0.000	Supported
H2b	ENC → ALP	0.026 ^ns.^	0.628	0.531	Not Supported
H2c	ENC → PSP	0.021 ^ns.^	0.491	0.624	Not Supported
H3a	SIV → ACP	0.276 ***	5.989	0.000	Supported
H3b	SIV → ALP	−0.008 ^ns.^	0.179	0.858	Not Supported
H3c	SIV → PSP	0.106 *	2.130	0.034	Supported
H4a	IDT → ACP	0.206 ***	4.698	0.000	Supported
H4b	IDT → ALP	0.144 **	2.700	0.007	Supported
H4c	IDT → PSP	0.084 ^ns.^	1.597	0.111	Not Supported
H5a	CFB → ACP	0.231 ***	4.422	0.000	Supported
H5b	CFB → ALP	0.138 ***	3.239	0.001	Supported
H5c	CFB → PSP	0.207 ***	4.331	0.000	Supported
H6a	SCB → ACP	0.585 ***	15.127	0.000	Supported
H6b	SCB → ALP	0.250 ***	4.352	0.000	Supported
H6c	SCB → PSP	−0.042 ^ns.^	0.839	0.402	Not Supported
H7a	STB → ACP	0.245 ***	4.455	0.000	Supported
H7b	STB → ALP	0.173 ***	3.335	0.001	Supported
H7c	STB → PSP	0.218 ***	4.584	0.000	Supported
H8a	ACP → BEV	0.297 ***	9.163	0.000	Supported
H8b	ALP → BEV	0.268 ***	6.198	0.000	Supported
H8c	PSP → BEV	0.399 ***	10.500	0.000	Supported

*** = *p*-value ≤ 0.001, ** = *p*-value ≤ 0.01, * = *p*-value ≤ 0.05, ns. = not-significant.

## Data Availability

The data are available upon request due to privacy restrictions.
